# Intensity of Psychoactive Substance Use Affects the Occurrence of Prodromal Symptoms of Psychosis

**DOI:** 10.3390/jcm13030760

**Published:** 2024-01-28

**Authors:** Gniewko Więckiewicz, Iga Florczyk, Maciej Stokłosa, Marta Jurga, Piotr Gorczyca, Magdalena Kotlicka-Antczak

**Affiliations:** 1 Department of Psychiatry, Faculty of Medical Sciences in Zabrze, Medical University of Silesia, 42-612 Tarnowskie Góry, Poland; 2 Department of Psychoprophylaxis, Faculty of Medical Sciences in Zabrze, Medical University of Silesia, 42-612 Tarnowskie Góry, Poland; 3 4th Military Teaching Hospital, 50-981 Wroclaw, Poland; 4 Child and Adolescent Psychiatry Clinic, Department of Psychiatry, Medical University of Lodz, 90-419 Lodz, Poland

**Keywords:** psychosis, psychoactive substance, drugs, addiction, schizophrenia, prodromal symptoms, DUDIT, PQ-B

## Abstract

Background: Psychosis is defined as a series of symptoms that impair the mind and lead to a kind of loss of reference to reality. Development of psychosis is usually preceded by the appearance of prodromal symptoms. Numerous attempts have been made to find out how psychoactive substances can influence the onset and development of psychotic disorders, but to date there are no studies that show a link between the onset of prodromal symptoms and the use of psychoactive substances. Methods: A survey consisting of epidemiological and demographic questions, the Drug Use Disorders Identification Test (DUDIT), and the Prodromal Questionnaire Brief Version (PQ-B) was conducted on social media among users of illegal psychoactive substances, covering 703 study participants. Results: A total of 39.8% of the respondents had been treated by a psychiatrist, and the most popular drugs used by respondents in their lifetime were tetrahydrocannabinol-containing products, MDMA, amphetamines, and LSD. A significant correlation was found between the DUDIT and the PQ-B values. Conclusions: Intensity of psychoactive substance use correlated positively with the risk of appearance and intensity of prodromal symptoms of psychosis. Early exposure to psychoactive substances increased the risk of heavy substance use in adulthood and led to more frequent prodromal states.

## 1. Introduction

According to the U.S. National Institutes of Health, psychosis is defined as a set of symptoms that impair the mind and lead to a kind of loss of reference to reality [1]. Other authors have stated that psychosis is a serious psychopathological condition characterized by a loss of contact with reality [2]. In up to two thirds of cases, the development of psychosis is preceded by the occurrence of so-called prodromal symptoms [3]. Around 25% of patients with prodromal symptoms go on to develop full-blown psychosis within 3 years. Recognizing prodromal symptoms is important as it allows for timely intervention, which can delay the development of a first psychotic episode [4]. The prodromal stage of psychosis represents the early phase of developing psychotic disorders, characterized by subtle and often non-specific symptoms that precede the onset of full-blown psychosis. CAARMS (Comprehensive Assessment of At Risk Mental States) is a valuable diagnostic tool for identifying and assessing these prodromal symptoms in seven different areas: 1. Positive symptoms: This area includes the presence of abnormal experiences or perceptions that deviate from typical reality, such as hallucinations or delusional thoughts. 2. Cognitive changes: In prodromal stages, there may be changes in cognitive functioning, including disturbances in attention, memory, and executive functions. 3. Emotional impairment: Individuals in the prodromal phase may experience changes in mood and affect, possibly characterized by increased anxiety, depression, or other emotional irregularities. 4. Negative changes: Negative symptoms include a deterioration in normal functioning, including social withdrawal, decreased motivation, and reduced expression of emotions. 5. Behavioral changes: Observable behavioral changes, such as increased irritability, unusual social interactions, or changes in daily activities, may indicate the onset of psychosis. 6. Motor/physical changes: Physical manifestations, such as changes in motor coordination or odd movements, may indicate the prodromal stage. 7. General psychopathology: This area includes a range of non-specific psychopathological symptoms that do not fit into the other categories and provides a broader perspective on the person’s mental state [5].

The onset of prepsychotic symptoms is a condition that prompts people to seek help from their general practitioner or relatives [6]. Due to the risk of the transformation of the prodromal stage into the development of psychosis, it is essential to educate health professionals about the occurrence of this type of disorder [7]. A descriptive model for prepsychotic symptoms was proposed by McGorry and includes four stages [8]. The first stage is associated with an increased risk of developing psychosis due to a family history of psychosis but with no current symptoms. Stage 1 represents the current conceptualization of the psychosis model by symptom severity—stage 1a for mild, non-specific symptoms and stage 1b for moderate, subthreshold symptoms. The remaining stages (2–4) cover the period from the first psychotic episode to long-term chronic illness [9]. An estimated 65% of individuals exhibiting prodromal symptoms do not progress to full psychosis, while those at highest risk of progressing to a full disorder are referred to as being at a clinically high risk of psychosis (CHR-P) [10].

Numerous attempts have been made to find out how psychoactive substances can influence the onset and development of psychotic disorders, including schizophrenia. One systematic review concluded that the tetrahydrocannabinol (THC) contained in cannabis may contribute to triggering the onset of symptoms in genetically predisposed individuals, as well as exacerbating symptoms and increasing the number and duration of psychiatric hospitalizations, while there is no causal relationship between THC use and the onset of schizophrenia [11]. Abuse of amphetamine and its derivatives can certainly produce acute psychotic symptoms, but there are no conclusive reports suggesting a possible link between the use of this substance and the subsequent development of schizophrenia; nevertheless, the manufacturing symptoms produced by amphetamine use may be a predictor of the development of primary psychosis [12]. Many of the substances used illicitly have been shown to have psychomimetic potential, but the features of psychotic production caused solely by substance abuse need to be distinguished from the development of schizophrenia [13]. About 30% of psychoses induced by psychoactive substances can develop into schizophrenia or bipolar affective disorder [14]. On the other hand, however, there are only a few reports in the literature of persistent psychotic symptoms among users of cannabis, methamphetamine, or cocaine after substance withdrawal [13].

There are no studies demonstrating a clear link between the appearance of prodromal symptoms and the use of psychoactive substances, but there are reports in the contemporary literature indicating the possibility of a more rapid transformation to full-blown psychosis in the users of these drugs [15]. For this reason, the present study sought to confirm the correlation between psychoactive substance use and the severity of prodromal symptoms.

## 2. Material and Methods

It is not easy to reach users of often illegal psychoactive substances, as the perception of drug use in society as a legal problem rather than a health problem can lead to respondents answering the questions incorrectly. Therefore, it was decided to recruit participants via social media, which aims for complete anonymity and is a proven method of collecting data for scientific publications in this group [16,17]. Data were collected over a 30-day period in November/December 2022. The survey was advertised on Facebook groups and pages related to drug use and on Instagram under hashtags related to psychoactive substance use. The survey was completely anonymous; the authors did not collect the IP addresses or email addresses of the respondents. The collected anonymized data were processed in accordance with the applicable data protection regulations of the Republic of Poland and the European Union. The study was exploratory, so no specific research questions were asked. Two inclusion criteria were used: age over or equal to 18 years and use of psychoactive substances. One exclusion criterion was used: diagnosed psychotic or bipolar disorder.

### 2.1. Survey

The survey was hosted on Google Forms, a user-friendly tool for the authors and respondents to create or submit surveys online. Before starting the survey, users had to confirm that they agreed to the data being shared and processed for research purposes. The survey consisted of the following elements:

− A separate questionnaire with epidemiological and demographic questions, such as age, education, marital status, and type, frequency, and amount of drug use.
− Drug Use Disorders Identification Test (DUDIT), a screening tool to identify psychoactive substance use disorders. It was developed to assess problems related to the use of drugs and medication, among other things. The self-report test consists of 11 questions designed to assess the various aspects of psychoactive substance use, such as frequency of use, patterns of use, negative consequences of drug use, attempts to reduce abuse, and other drug-use behaviors. A total score is calculated from the answers to the questions, which is then interpreted in relation to the predisposition to drug abuse. Questions 1–9 can be scored with 0–4 points, while questions 10–11 can be scored with 0, 2, or 4 points. The maximum number of points is 44. The cut-off points in the DUDIT test are gender-specific. This means that if a man scores more than 6 points or a woman scores more than 2 points, there is a probability of substance abuse or harmful use of psychoactive substances. With a score of over 25 for both sexes, there is a high probability of a risk of addiction to several substances. To obtain an accurate diagnosis, it is recommended to complement the initial assessment with the DUDIT with another clinical tool; however, according to the available literature, this test has adequate psychometric parameters, making it a valuable screening tool for detecting psychoactive substance abuse tendencies and assessing the severity of problematic psychoactive substance use [18].
− The Prodromal Questionnaire Short Version (PQ-B), a screening instrument used in psychiatry to identify prodromal or precursor symptoms associated with schizophrenia and other psychotic disorders. The PQ-B is a shortened version of the original Prodromal Questionnaire (PQ), which consists of 92 items. In the PQ-B, only the items on positive symptoms are retained, with a total of 21 questions from which respondents can obtain a total score (yes/no) of a maximum of 21 points or a distress score of a maximum of 105 points, which is preferred to maximize sensitivity and specificity. It is designed to capture prodromal symptoms, such as persecutory delusions, ideas of reference, perceptual disturbances, unusual ideas, and unusual beliefs. In addition, there are questions on mistrust, pride, and disorganized communication, as well as social and academic/professional functioning [19]. The questions are designed to assess the frequency and severity of these symptoms over the past few months. A distress score or a 5-point Likert scale was used to count the responses, where respondents were asked to respond to the given statements from “strongly disagree” (0) to “strongly agree” (5). If the respondent reaches a score of six or more, it is recommended that their disorder is assessed by a psychiatrist, e.g., using the Structured Interview for Psychosis Risk Syndromes (SIPS) [20,21]. The purpose of the PQ-B is to identify individuals at risk of developing psychosis or schizophrenia in order to offer them a more in-depth assessment and appropriate follow-up. It is a relatively simple and suitable instrument for screening psychosis-like experiences [22].

Both diagnostic instruments (DUDIT and PQ-B) have been used in their validated and widely accepted Polish versions.

### 2.2. Statistics

STATISTICA 13.3 (StatSoft, Krakow, Poland) was used to analyze the collected data. The Shapiro–Wilk Test was used to assess the normality of the distribution of the variables. The Pearson’s chi-squared test was used to compare the qualitative variables. For quantitative variables, the Mann–Whitney U test was used to compare two independent groups, while the Kruskal–Wallis test was used to compare multiple independent groups. Spearman’s rank correlation was used to examine the correlation of the linear variables. To assess the parameters influencing the PQ-B scale, we performed univariate and multivariate linear regression analysis with elimination of the backward variables. The significance level was set at *p* < 0.05, which was then corrected using the False Discovery Rate (FDR).

## 3. Results

### 3.1. Characteristics

#### 3.1.1. Group Characteristics

A total of 748 completed questionnaires were collected, of which 45 questionnaires were rejected (one was incorrectly completed; 44 met the exclusion criteria). Table 1 shows the characteristics of the study sample. No significant differences were found between the genders in terms of age, place of residence, or education. Statistically significant differences between the genders were found with regard to sexual orientation, work situation, monthly income, and relationship status.

#### 3.1.2. Characteristics of Drug Consumption

The vast majority of respondents consumed alcohol, whereby there was no gender difference in the fact of consumption or the frequency of consumption. All respondents consumed drugs other than alcohol. There were no gender differences in the amount of types of drugs consumed, the age of first contact with psychoactive substances, or nicotine use, as shown in Table 2.

#### 3.1.3. Types of Drugs Taken

The spectrum of psychoactive substance use by respondents is shown in Table 3 and Table 4 in relation to their lifetime and the past year, respectively. The most popular drugs used in the lifetime of more than half of the respondents were THC-containing products, MDMA, amphetamines, and LSD. In the past year, the most commonly used drugs were THC, 3,4-methylenedioxymethamphetamine (MDMA), hallucinogenic mushrooms, amphetamine, and lysergic acid diethylamide (LSD).

#### 3.1.4. Drug-Taking Patterns

Table 5 contains data on the respondents’ drug use. Significant gender differences were evident in the most frequent accompanier to drug use (χ^2^ = 37.8; *p* < 0.0001; chi-squared test), while no such correlations were observed in the frequency of use of psychoactive substances, the source of these substances, testing their quality, the amount spent monthly on drugs, education in this area, problems with the law or family responsibilities, or the use of specialized help. Of note is the number of men who use drugs more than ten times a month, or the fact that half of the respondents do not measure the dose of the drugs they take. Men are significantly more likely to measure the dose of the substances they take (χ^2^ = 19.5; *p* = 0.003; chi-squared test).

#### 3.1.5. Survey on Psychiatric Treatment among the Interviewees

In the study group, we observed significantly more frequent psychiatric treatment among men (χ^2^ = 12.0; *p* = 0.003; chi-squared test). A total of 35% of respondents who had received psychiatric treatment had attempted suicide in the past. No gender-specific differences were found for the other questions on psychiatric treatment. Detailed data can be found in Table 6.

### 3.2. Analysis of the Relationship between Psychoactive Substance Use and Prodromal Symptoms

#### 3.2.1. DUDIT vs. PQ-B

Table 7 shows the distribution of the groups with clinical relevance and the average values on the DUDIT scale between the genders. Significant gender differences were observed in the DUDIT cut-off point, with significantly more females in the group with drug-related problems (χ^2^ = 12.0; *p* < 0.0001; chi-squared test). Similarly, Table 8 shows the distribution of the median scores and groups with clinical relevance on the PQ-B subscales between the genders. There was no significant difference observed in PQ-B total score; however, in distress score, there was a significant difference found (H = 10.1; *p* = 0.006; Kruskal–Wallis H test), and in post-hoc multiple comparisons there was a significant difference only between ‘male’ and ‘female’ (z = 2.96; *p* = 0.009).

A significant correlation was found between the DUDIT and the scores on both PQ-B subscales: a positive relationship for both frequency (r = 0.15; *p* < 0.0001) and distress (r = 0.17; *p* < 0.0001).

#### 3.2.2. DUDIT Cut-off Groups Compared with the PQ-B

There was a difference between the DUDIT cut-off groups on the PQ-B total score (H = 11.2; *p* < 0.01; Kruskal–Wallis H test), and in the post-hoc multiple comparisons, there was only a borderline significant difference between the groups: ‘below cut-off’ and ‘drug-related problems’ (z = 2.65; *p* = 0.048). The detailed group means are shown in Table 9.

In a direct analysis of the two groups above the cut-off of the DUDIT scale, the ‘highly probable dependence’ group showed a higher frequency of prodromal symptoms in PQ-B compared with the other respondents (U Mann-Whitney test; z = −3.566; *p* < 0.001) and a higher score on the PQ-B distress scale (U Mann-Whitney test; z = −3.16; *p* = 0.0015). We did not find the same correlation in the ‘drug-related problems’ group (U Mann-Whitney test; NS).

#### 3.2.3. Age Versus PQ-B Value

Younger respondents exhibited prodromal symptoms significantly more frequently and had a higher score on the PQ-B Distress Scale (r = −0.24 and r = −0.25 respectively; *p* < 0.0001).

#### 3.2.4. Age of Onset of Drug Use vs. PQ-B Score

Earlier exposure to psychoactive substances correlated with more types of drugs taken (r = −0.14; *p* < 0.0001), more frequent heavy drug use (r = −0.1; *p* = 0.007), greater psychological or physical harm caused by drug use (r = −0.11; *p* = 0.004), and more frequent symptoms that distress the respondent (r = −0.09; *p* = 0.012). It was also found that those with highly probable dependence had their first drug use significantly earlier (z = 2.1; *p* = 0.035), but there is no such association in the group with only high-risk drug-related problems. In addition, former drug users more frequently showed prodromal symptoms on the PQ-B scale (r = 0.16; *p* < 0.0001) and had a higher score on the PQ-B distress scale (r = 0.17; *p* < 0.0001).

#### 3.2.5. Number of Types of Drugs Taken

There is a significant correlation between the number of types of drugs taken and the result of the PQ-B scale for both the frequency and the stress scale—in each case in relation to the number of types of drugs taken in the past 12 months (PQ-B: frequency—r = 0.15; *p* < 0.0001; PQ-B: distress scale—r = 0.11; *p* = −0.004), as well as during their lifetime (PQ-B: frequency—r = 0.13; *p* < 0.0005; PQ-B: distress scale—r = 0.09; *p* = −0.02).

#### 3.2.6. Psychiatric Treatment vs. PQ-B

No significantly higher incidence of prodromal symptoms was observed in the group with psychiatric treatment.

#### 3.2.7. Declared Alcohol Consumption vs. PQ-B

There was no correlation between the frequency of alcohol consumption and the occurrence of prodromal symptoms.

### 3.3. Linear Regression Analysis for the PQ-B Scale

Univariate and multivariate linear regression analyses were performed for the subscales of the PQ-B scale: ‘total score’ and ‘distress score’. In the univariate analysis for the ‘total score’, the 16 variables were proved to be statistically significant. The variables from the univariate analysis with *p* < 0.05 were included in the multivariate analysis. As a result, it was found that the significant factors independently influencing the ‘total score’ subscale of the PQ-B scale were gender, age, relationship life, size of residence, education level, and the number of types of drugs taken in their lifetime and in the past year. The results are presented in Table 10.

In the univariate analysis for ‘distress score’, 17 variables were classified as statistically significant. The variables from the univariate analysis with *p* < 0.05 were included in the multivariate analysis. In the constructed multivariate model, significant factors were found that independently influenced the result of the ‘distress score’ subscale of the PQ-B: gender, age, relationship status, education level, number of drugs taken in their lifetime, question 3 of the DUDIT questionnaire, DUDIT score, and psychiatric treatment in the past. The results are shown in Table 11.

## 4. Discussion

Drug use can significantly impair brain function and increase the risk of mental health problems, including psychosis. Susceptibility to psychosis is often the result of complex genetic and environmental interactions. Drug use can act as a trigger, particularly in people who already have a genetic predisposition to the disorder. In the context of the development of psychosis, it is important to take a holistic approach to mental health assessment, taking into account a variety of factors, including psychoactive substance use. This work is part of this trend. Its findings can be used to develop strategies for effective risk management by providing appropriate support to people at risk of developing psychosis.

In this study, no significant gender differences were observed regarding the use of psychoactive substances or nicotine, and there were also no significant differences between men and women regarding the age of their first contact with drugs. Studies over the past decade have shown that men are significantly more likely to use drugs than women, but the gender gap is gradually closing [23].

In the group of individuals who met the criteria for a high likelihood of developing a dependence on psychoactive substances, prodromal symptoms and associated problems were statistically more common than in individuals who did not exhibit substance abuse tendencies. Previous studies have confirmed the association between cannabinoid abuse and predisposition to the development of the prodromal symptoms of schizophrenia and psychotic disorders [24]. Similar data have been obtained in recent studies conducted in a group of adolescents who appear to be at relatively high risk of developing prodromal symptoms [25]. Studies also suggest that an increased risk of developing schizophrenia may also apply to methamphetamine abusers [26]. Data on other psychoactive substances and the possible development of psychosis as a result of their abuse remain limited [15].

The age of initiation of drug use is also a variable that significantly modulates the risk of developing prodromal symptoms, but also influences higher distress scores. This has also been confirmed in other reports hypothesizing that younger individuals among psychoactive substance users may represent a subclinical risk group for psychosis [27]. Effective addiction prevention and psychoeducation among young people about the relationship between substance use and the possible occurrence of prodromal symptoms is a key element in the prevention of psychotic disorders. Awareness of this connection can provide young people with important information about the risks associated with substance abuse, while also pointing out that such activities can be a predisposing factor for the development of early symptoms of psychosis. Incorporating these aspects into educational programs can not only help to reduce the risk of addiction, but also to identify and intervene earlier in psychotic prodromal states, which in turn can significantly impact the mental health of the younger generation.

There was a clear correlation between the age at which psychoactive substance use began and the effects on an individual’s health and behavior. The lower the stated age for starting to experiment with psychoactive substances, the higher the risk of damage to the individual’s mental and physical health, of trying more psychoactive substances, and of being heavily influenced by them more often. A similar relationship was observed in a study conducted in Kuwait, which concluded that an earlier age of first contact with drugs led to more frequent drug use and a higher likelihood of developing an addiction [28]. The earlier the respondents turned to drugs, the more anxiety-inducing symptoms were observed in their environment. Scientific reports have described a link between the abuse of amphetamines, hallucinogens, cocaine, and heroin and symptoms of increased anxiety and anxiety disorders [29].

The more different types of drugs the respondents used, the higher the likelihood that they developed prodromal symptoms. Previous studies have argued that the use of cannabinoids, but also of other psychoactive substances, can lead to earlier development of prodromal symptoms and psychosis in susceptible individuals, and that this effect is related to the dose of the drugs used [30]. This study did not show that the simultaneous use of alcohol and psychoactive substances could have an effect on the development of prodromal symptoms; however, previous studies have drawn different conclusions—in a study conducted in a group of adolescents, it was shown that alcohol in combination with cannabinoid use significantly increased the risk of developing psychotic disorders [25].

The occurrence of prodromal symptoms is also influenced by gender, age, relationship status, education level, the size of the place of residence, and the number of psychoactive substances taken in the course of their lifetime and in the past year. The protective factors for psychosis identified in studies include a higher intelligence quotient, family support, and higher social skills, as well as personality traits such as extraversion, openness, agreeableness, and conscientiousness [31,32]. This information can also be used by clinicians in psychoeducating the patient, to guide the therapeutic process and reinforce positive behaviors that can slow the development of psychosis [33]. It is worth noting that some symptoms that are considered prodromal and are assessed using the PQ-B tool may occur under the influence of psychoactive substances; however, an important aspect is that the respondents were accurately instructed according to the PQ-B guidelines, noting that the questions refer to sensations in the past month and exclude the influence of substances related to an episode of intoxication. Such clarification is crucial for the accurate interpretation of the results, as it eliminates possible bias related to substance use and allows the focus on relevant prodromal symptoms associated with the risk of developing psychotic disorders.

### Limitations

Anonymous surveys conducted over the Internet have their unique advantages, but they also come with certain limitations, such as the lack of certainty about the identity of respondents, as the risk of providing false data cannot be ruled out, which can affect the quality of the information collected. Internet surveys can tend to attract certain groups of people, which leads to problems with the representativeness of the sample. People without internet access, older people, or people of lower socio-economic status may be under-represented. Respondents may interpret the worded questions differently, which can lead to incorrect answers. The absence of a researcher to clarify respondents’ doubts may affect the quality of the data collected. Respondents’ answers can be influenced by technical problems, such as problems with the internet connection and differences in the devices or the software used. A survey is an effective tool for data collection due to the greater openness of the participants, especially with regard to anonymity, which is particularly important when it comes to drugs.

## 5. Conclusions

1. The intensity of consumption of psychoactive substances correlates positively with the risk of occurrence and the intensity of prodromal symptoms of psychosis.
2. Alcohol consumption has no influence on prodromal symptoms in drug users.
3. Early exposure to psychoactive substances increases the risk of heavy substance use in adulthood and leads to more frequent prodromal symptoms.

## Figures and Tables

**Table 1:** Study group characteristics (M—male; F—female; NS—non significant; (χ)^2^—Chi-squared test; *—Kruskal–Wallis H test; ** After correction using FDR).

			All	M	F	Other	*p* Value
	*n*		703	360	331	12	
	%		100.0%	51.2%	47.1%	1.7%	
	Age [median]		25	26	25	27.5	NS *
Sexual orientation:	Heterosexual		76.5%	84.7%	70.1%	8.3%	<0.0001 (χ)^2^
	Bisexual		14.9%	4.7%	24.8%	50.0%	
	Homosexual		6.1%	9.7%	2.1%	8.3%	
	Other		2.4%	0.8%	3.0%	33.3%	
Size of residence:	Village		9.8%	12.8%	6.6%	8.3%	NS ** (χ)^2^
	<50 k		12.2%	13.1%	11.8%	0.0%	
	50–200 k		16.2%	15.3%	17.8%	0.0%	
	>200 k		61.7%	58.9%	63.7%	91.7%	
Education:	Basic		1.4%	1.7%	1.2%	0.0%	NS (χ)^2^
	Middle school		3.3%	4.7%	1.8%	0.0%	
	Medium		47.8%	51.7%	43.8%	41.7%	
	Higher Bachelor’s degree		23.3%	19.2%	27.8%	25.0%	
	Higher Master’s degree		22.5%	21.4%	23.6%	25.0%	
	Doctorate or higher degree/title		1.7%	1.4%	1.8%	8.3%	
Professional situation:	Full-time employee		44.4%	44.7%	44.1%	41.7%	0.003 (χ)^2^
	Entrepreneur		12.9%	16.9%	8.5%	16.7%	
	Casual worker		3.1%	3.6%	2.7%	0.0%	
	Student	Unemployed	10.4%	10.3%	9.7%	33.3%	
		Working	19.3%	15.6%	24.2%	0.0%	
	Student	Unemployed	3.8%	4.2%	3.6%	0.0%	
		Working	2.8%	3.3%	2.4%	0.0%	
	Unemployment		2.8%	1.1%	4.5%	8.3%	
	Retirement or pension		0.3%	0.3%	0.3%	0.0%	
Net monthly earnings [Polish zloty]:	<2000		11.1%	8.0%	14.5%	8.3%	<0.0001 (χ)^2^
	2000–3000		10.5%	6.9%	14.8%	0.0%	
	3000–4000		17.8%	15.6%	20.8%	0.0%	
	4000–5000		12.2%	11.9%	12.1%	25.0%	
	>5000		31.0%	41.4%	20.2%	16.7%	
	Not applicable		17.4%	16.1%	17.5%	50.0%	
Relationship status:	In a formal relationship (marriage)		10.4%	9.2%	11.5%	16.7%	<0.0001 (χ)^2^
	In an informal relationship (including engagement)		49.9%	42.8%	58.6%	25.0%	
	Single/single		34.0%	42.8%	25.4%	8.3%	
	In a polyamorous/polygamous relationship		2.4%	1.9%	1.5%	41.7%	
	Other		1.7%	1.7%	1.5%	8.3%	

**Table 2:** General characteristics of alcohol, nicotine, and drugs used among respondents (NS—non significant; (χ)^2^—Chi-squared test; *—Kruskal–Wallis H test; ** After correction using FDR; M—male; F—female).

			All	M	F	Other	*p* Value (χ^2^/*)
	*n*		703	360	331	12	
	%		100.0%	51.2%	47.1%	1.7%	
Do you consume alcohol?	Yes		82.7%	81.1%	84.6%	75.0%	NS
	No		17.4%	18.9%	15.4%	25.0%	
How often do you consume alcohol?	Once every few months or less frequently		7.4%	5.1%	9.6%	11.1%	NS **
	1×/month		10.3%	8.9%	11.8%	11.1%	
	2–3×/month		26.2%	24.7%	27.5%	33.3%	
	4–5×/month		21.9%	22.3%	21.8%	11.1%	
	6–8×/month		14.3%	13.0%	15.7%	11.1%	
	9–10×/month		7.6%	8.2%	6.8%	11.1%	
	>10×/month		12.4%	17.8%	6.8%	11.1%	
Are you taking nicotine?	Yes	Classic cigarettes	26.7%	25.3%	28.7%	16.7%	NS
		e-cigarettes	15.9%	16.6%	15.7%	0.0%	
		Tobacco vaporization	16.4%	14.2%	18.1%	33.3%	
		other	5.1%	7.22%	3.02%	0.0%	
	No		35.9%	36.7%	34.4%	50.0%	
Do you use psychoactive substances other than alcohol?	No		0%	0%	0%	0%	-
	Yes		100%	100%	100%	100%	
How many types of drugs were used:	Over a lifetime		6	6	5	5	NS *
	Over the past year		3	3	3	4	NS *
Age of first exposure to psychoactive substances			16	16	16	16	NS *

**Table 3:** Characteristics of drugs used by respondents throughout their lives (THC—tetrahydrocannabinol; MDMA—3,4-methylenedioxymethamphetamine; LSD—lysergic acid diethylamide; M—male; F—female).

		All	M	F	Other
	*n*	703	360	331	12
	%	100.0%	51.2%	47.1%	1.7%
What psychoactive substances have you used throughout your life?					
THC:	No	1.8%	1.1%	2.7%	0.0%
	Yes	98.2%	98.9%	97.3%	100.0%
Amphetamine:	No	42.2%	42.5%	42.0%	41.7%
	Yes	57.8%	57.5%	58.0%	58.3%
Methamphetamine:	No	82.1%	80.8%	83.1%	91.7%
	Yes	17.9%	19.2%	16.9%	8.3%
MDMA:	No	26.9%	28.6%	25.1%	25.0%
	Yes	73.1%	71.4%	74.9%	75.0%
Cocaine:	No	57.0%	57.2%	56.5%	66.7%
	Yes	43.0%	42.8%	43.5%	33.3%
Mephedrone:	No	53.1%	52.8%	53.2%	58.3%
	Yes	46.9%	47.2%	46.8%	41.7%
Morphine:	No	94.3%	94.2%	94.6%	91.7%
	Yes	5.7%	5.8%	5.4%	8.3%
Fentanyl:	No	98.3%	97.8%	98.8%	100.0%
	Yes	1.7%	2.2%	1.2%	0.0%
Heroin:	No	98.2%	98.3%	97.9%	100.0%
	Yes	1.8%	1.7%	2.1%	0.0%
Other opioids:	No	83.8%	83.1%	84.9%	75.0%
	Yes	16.2%	16.9%	15.1%	25.0%
LSD:	No	48.6%	44.4%	52.9%	58.3%
	Yes	51.4%	55.6%	47.1%	41.7%
Hallucinogenic mushrooms:	No	45.5%	37.5%	54.4%	41.7%
	Yes	54.5%	62.5%	45.6%	58.3%
Synthetic cannabinoids:	No	87.6%	85.8%	89.4%	91.7%
	Yes	12.4%	14.2%	10.6%	8.3%
Dimethyltryptamine (DMT):	No	87.2%	86.1%	88.8%	75.0%
	Yes	12.8%	13.9%	11.2%	25.0%
Ketamine:	No	77.5%	77.5%	77.9%	66.7%
	Yes	22.5%	22.5%	22.1%	33.3%
Benzodiazepines:	No	71.0%	73.1%	69.5%	50.0%
	Yes	29.0%	26.9%	30.5%	50.0%
Benzofurans:	No	97.9%	97.2%	98.5%	100.0%
	Yes	2.1%	2.8%	1.5%	0.0%
Mescaline:	No	98.7%	97.8%	99.7%	100.0%
	Yes	1.3%	2.2%	0.3%	0.0%
Aminorex:	No	99.7%	99.4%	100.0%	100.0%
	Yes	0.3%	0.6%	0.0%	0.0%
Alkyl nitrites (“poppers”):	No	88.3%	87.2%	90.0%	75.0%
	Yes	11.7%	12.8%	10.0%	25.0%
Other:	No	81.2%	80.8%	83.1%	41.7%
	Yes	18.8%	19.2%	16.9%	58.3%

**Table 4:** Characteristics of drugs used by respondents in the past year (THC—tetrahydrocannabinol; MDMA—3,4-methylenedioxymethamphetamine; LSD—lysergic acid diethylamide; M—male; F—female).

		All	M	F	Other
	*n*	703	360	331	12
	%	100.0%	51.2%	47.1%	1.7%
What psychoactive substances have you used in the past year?					
THC:	No	12.8%	10.8%	15.4%	0%
	Yes	87.2%	89.2%	84.6%	100.0%
Amphetamine:	No	68.0%	70.6%	65.6%	58.3%
	Yes	32.0%	29.4%	34.4%	41.7%
Methamphetamine:	No	91.9%	91.1%	92.4%	100.0%
	Yes	8.1%	8.9%	7.6%	0%
MDMA:	No	51.2%	50.8%	51.1%	66.7%
	Yes	48.8%	49.2%	48.9%	33.3%
Cocaine:	No	76.8%	78.1%	75.2%	83.3%
	Yes	23.2%	21.9%	24.8%	16.7%
Mephedrone:	No	70.8%	71.1%	70.4%	75.0%
	Yes	29.2%	28.9%	29.6%	25.0%
Morphine:	No	98.4%	98.6%	98.2%	100.0%
	Yes	1.6%	1.4%	1.8%	0%
Fentanyl:	No	99.1%	98.9%	99.4%	100.0%
	Yes	0.9%	1.1%	0.6%	0%
Heroin:	No	99.7%	100.0%	99.4%	100.0%
	Yes	0.3%	0%	0.6%	0%
Other opioids:	No	93.7%	93.3%	94.6%	83.3%
	Yes	6.3%	6.7%	5.4%	16.7%
LSD:	No	68.8%	66.4%	71.6%	66.7%
	Yes	31.2%	33.6%	28.4%	33.3%
Hallucinogenic mushrooms:	No	62.7%	55.8%	70.4%	58.3%
	Yes	37.3%	44.2%	29.6%	41.7%
Synthetic cannabinoids:	No	98.2%	98.3%	97.9%	100.0%
	Yes	1.8%	1.7%	2.1%	0%
Dimethyltryptamine (DMT):	No	94.7%	93.6%	96.1%	91.7%
	Yes	5.3%	6.4%	3.9%	8.3%
Ketamine:	No	89.6%	90.6%	88.8%	83.3%
	Yes	10.4%	9.4%	11.2%	16.7%
Benzodiazepines:	No	86.2%	86.7%	86.7%	58.3%
	Yes	13.8%	13.3%	13.3%	41.7%
Benzofurans:	No	99.1%	98.9%	99.4%	100.0%
	Yes	0.9%	1.1%	0.6%	0%
Mescaline:	No	99.9%	99.7%	100.0%	100.0%
	Yes	0.1%	0.3%	0.0%	0.0%
Aminorex:	No	100%	100%	100%	100%
	Yes	0%	0%	0%	0%
Alkyl nitrites (“poppers”):	No	96.3%	96.9%	96.1%	83.3%
	Yes	3.7%	3.1%	3.9%	16.7%
Other:	No	93.2%	93.6%	93.7%	66.7%
	Yes	6.8%	6.4%	6.3%	33.3%

**Table 5:** Characteristics of the respondents’ drug-use patterns (NS—non significant; (χ)^2^—Chi-squared test; M—male; F—female).

		All	M	F	Other	*p* Value (χ^2^)
	*n*	703	360	331	12	
	%	100.0%	51.2%	47.1%	1.7%	
How often do you use psychoactive substances?	Once every few months or less frequently	34.3%	30.8%	37.2%	58.3%	NS
	1×/month	14.8%	13.6%	16.0%	16.7%	
	2–3×/month	13.1%	13.1%	13.3%	8.3%	
	4–5×/month	6.8%	6.7%	7.3%	0.0%	
	6–8×/month	5.8%	6.1%	5.7%	0.0%	
	9–10×/month	3.7%	4.7%	2.7%	0.0%	
	>10×/month	21.5%	25.0%	17.8%	16.7%	
With whom do you most often use psychoactive substances?	With friends	53.5%	56.9%	49.2%	66.7%	<0.0001
	With a partner/partner	22.5%	15.6%	30.2%	16.7%	
	By yourself	22.0%	25.3%	19.0%	8.3%	
	With family	0.6%	0.3%	0.6%	8.3%	
	Other than the above	1.4%	1.9%	0.9%	0.0%	
Where do you most often use psychoactive substances?	At home	49.4%	46.4%	52.6%	50.0%	NS
	At someone’s home	24.6%	26.4%	23.0%	16.7%	
	At music festivals	5.0%	4.2%	5.4%	16.7%	
	Outdoors (forests, meadows)	10.4%	12.2%	8.8%	0.0%	
	In bars	0.9%	1.1%	0.6%	0.0%	
	In clubs	9.8%	9.7%	9.7%	16.7%	
Where do you most often obtain your psychoactive substances?	From friends	59.5%	57.2%	61.9%	58.3%	NS
	From a dealer/strangers at music festivals/in clubs or bars	17.5%	15.8%	19.0%	25.0%	
	In the darknet	5.1%	5.6%	4.8%	0.0%	
	Other options not included in the survey	17.9%	21.4%	14.2%	16.7%	
Do you test your psychoactive substances with colorimetric reagents or send them to a lab for testing?	No, never	79.2%	77.5%	81.6%	66.7%	NS
	Sometimes	16.5%	18.6%	13.9%	25.0%	
	Yes, always	4.3%	3.9%	4.5%	8.3%	
How much do you spend on psychoactive substances in a month?	I don’t spend money, I get it from someone	16.9%	15.0%	19.0%	16.7%	NS
	up to 100 PLN	40.0%	36.1%	43.8%	50.0%	
	100–200 PLN	15.2%	18.1%	11.8%	25.0%	
	200–300 PLN	9.8%	10.6%	9.4%	0%	
	300–400 PLN	8.0%	8.3%	7.9%	0%	
	more than 500 PLN	10.1%	11.9%	8.2%	8.3%	
Do you measure the doses of your psychoactive substances?	Never	7.0%	4.7%	9.1%	16.7%	0.003
	Mostly “by eye”	42.4%	40.0%	45.3%	33.3%	
	Sometimes	14.8%	16.1%	12.4%	41.7%	
	Yes, always	35.8%	39.2%	33.2%	8.3%	
Do you educate yourself about the safety and risks of using psychoactive substances?	Yes	92.3%	92.2%	92.4%	91.7%	NS
	No	7.7%	7.8%	7.6%	8.3%	
Have you ever been or are you in trouble with the law because of your use of psychoactive substances?	Yes	8.8%	10.8%	6.6%	8.3%	NS
	No	91.2%	89.2%	93.4%	91.7%	
Have you ever neglected your daily responsibilities (work, study, family life) by taking psychoactive substances?	No, never	50.1%	49.7%	49.8%	66.7%	NS
	Yes, once	10.4%	9.2%	11.5%	16.7%	
	Yes, several times	34.7%	37.5%	32.3%	16.7%	
	Yes, it happens to me often	4.8%	3.6%	6.3%	0%	
Have you thought or have you thought about seeking professional help for substance abuse?	Yes	18.1%	17.8%	18.7%	8.3%	NS
	No	81.9%	82.2%	81.3%	91.7%	
Have you ever sought medical help shortly after taking psychoactive substances? (e.g., acute poisoning)	Yes	9.1%	6.9%	11.5%	8.3%	NS
	No	90.9%	93.1%	88.5%	91.7%	

**Table 6:** Characteristics of the psychiatric treatment of the respondents (M—male; F—female; NS—non significant; (χ)^2^—Chi-squared test).

		All	M	F	Other	*p* Value (χ^2^)
	*n*	703	360	331	12	
	%	100.0%	51.2%	47.1%	1.7%	
Have you ever been treated by a psychiatrist or in a psychiatric unit?	Yes	39.8%	33.6%	46.2%	50.0%	0.003
	No	60.2%	66.4%	53.8%	50.0%	
For what reason was there psychiatric treatment? (*n* = 280)	Depressive disorders	56.1%	57.9%	55.6%	33.3%	NS
	Addiction treatment	4.6%	3.3%	5.9%	0%	
	Insomnia	2.5%	4.1%	1.3%	0%	
	Anxiety disorders	18.6%	19.8%	17.0%	33.3%	
	Personality disorders	6.1%	5.0%	6.5%	16.7%	
	ADHD	6.1%	4.1%	7.2%	16.7%	
	Other	3.9%	3.3%	4.6%	0%	
	Adaptive disorders	2.1%	2.5%	2.0%	0%	
Have there ever been any suicide attempts? (*n* = 280)	No	64.9%	73.3%	59.8%	60.0%	NS
	Yes, once	16.5%	11.4%	19.5%	20.0%	
	Yes, several times	18.6%	15.2%	20.7%	20.0%	
Did psychiatric treatment precede the onset of psychoactive substance use? (*n* = 280)	No, I went to a psychiatrist after I started using psychoactive substances	68.1%	71.4%	64.6%	90.0%	NS
	Yes, I started using psychoactive substances after my first visit to a psychiatrist	31.9%	28.6%	35.4%	10.0%	
Did the psychiatrist ask about the psychoactive substances used during the interview collection? (*n* = 280)	Yes	74.2%	78.1%	70.1%	100.0%	NS
	No	25.8%	21.9%	29.9%	0%	
When going to a psychiatrist, would you tell the truth about the psychoactive substances used? (*n* = 280)	Yes, always	35.0%	40.6%	29.3%	25.0%	NS
	Rather yes, if the doctor inspired my confidence	52.3%	49.4%	55.3%	58.3%	
	No—for other reasons	1.7%	1.1%	2.4%	0%	
	No—I don’t want to have anything “in the papers”	3.7%	2.5%	4.8%	8.3%	
	No—I’m afraid of the legal consequences	4.1%	4.2%	4.2%	0%	
	No—I’m afraid of being judged	3.1%	2.2%	3.9%	8.3%	
Would you answer the same questions if you received a paper survey?	Yes	73.5%	73.9%	72.8%	83.3%	NS
	No—the internet provides a sense of anonymity	14.8%	15.0%	14.8%	8.3%	
	No—filing online is more convenient	10.5%	9.7%	11.5%	8.3%	
	No—for other reasons	1.1%	1.4%	0.9%	0%	

**Table 7:** Distribution of groups with clinical significance and mean DUDIT scale scores among the genders (DUDIT—Drug Use Disorders Identification Test; M—male; F—female; NS—non significant; (χ)^2^—Chi-squared test; *—Kruskal–Wallis H test).

			All	M	F	Other	*p* Value
		*n*	703	360	331	12	
		%	100.0%	51.2%	47.1%	1.7%	
no drug use			1.6%	0.8%	2.1%	8.3%	*p* < 0.0001 (χ)^2^
DUDIT 0–44 pts	1	below cut-off	18.9%	31.9%	5.1%	8.3%	
	2	M: ≥6 K: ≥2 (drug-related problems)	75.4%	63.3%	88.2%	83.3%	
	3	≥25 (highly probable dependent)	4.1%	3.9%	4.5%	0%	
		median	7	8	7	4.5	NS *

**Table 8:** Distribution of mean scores and groups with clinical significance on the PQ-B scale among the genders (PQ-B—Prodromal Questionnaire–Brief; M—male; F—female; UHR—ultra high risk; NS—non significant; (χ)^2^—Chi-squared test; *—Kruskal–Wallis H test).

			All	M	F	Other	*p* Value
		*n*	703	360	331	12	
		%	100.0%	51.2%	47.1%	1.7%	
PQ-B scale	total score 0–21 pts	below cut-off	31.4%	32.8%	29.9%	33.3%	NS
		≥2 pct UHR	68.6%	67.2%	70.1%	66.7%	
		median	4	4	5	5	NS *
	distress score 0–105 pts	below cut-off	31.4%	33.9%	28.4%	41.7%	NS
		≥6 pct UHR	68.6%	66.1%	71.6%	58.3%	
		median	11	10 ***	13 ***	9	0.006 *

*** Post-hoc test—significant difference M vs. F.

**Table 9:** Distribution of PQ-B subscale score median among clinical groups on the DUDIT scale (M—male; F—female; DUDIT—Drug Use Disorders Identification Test; NS—non significant; (χ)^2^—Chi-squared test; *—Kruskal–Wallis H test; ** After correction using FDR).

		All		DUDIT Scale 0–44 pts			*p* Value *
			0	1	2	3	
			No Drugs	Below Cut-Off	M: ≥6 F: ≥2 (Drug-Related Problems)	≥25 (Highly Probable Dependence)	
PQ-B scale [median]	total score	4	3	3 ***	4 ***	5	0.01
	distress score	11	7	7	11	11	NS **

*** Post-hoc test—significant difference between groups: ‘below cut-off’ and ‘drug-related problems’.

**Table 10:** Univariate and multivariate analysis for parameters affecting the PQ-B subscale ‘total score’. (β—β coefficient; SE—standard errors for coefficients; t—t value; *p*—*p* value; NS—non significant).

		Univariate Analysis				Multivariate Analysis			
		β	SE	t	*p*	β	SE	t	*p*
Sociodemographic									
Gender		−0.72	0.34	−2.16	0.031 *	−1.07	0.33	−3.30	0.001 *
Age		−0.14	0.02	−5.73	<0.0001 *	−0.10	0.03	−3.84	<0.0001 *
Heterosexual/Non-heterosexual		−0.93	0.39	−2.39	0.017 *				NS
In relationship		−1.54	0.34	−4.54	<0.0001 *	−1.14	0.34	−3.37	0.0008 *
Size of city of residence		−0.37	0.16	−2.27	0.024 *	−0.33	0.16	−2.04	0.04 *
Level of education		−1.50	0.28	−5.34	<0.0001 *	−0.86	0.30	−2.85	0.005 *
Earnings		−0.48	0.09	−5.46	<0.0001 *				NS
**Psychoactive substance**									
Frequency of alcohol consumption		0.14	0.11	1.31	0.191				
Age of first drug usage		−0.02	0.02	−1.38	0.169				
Amount of types of drugs taken	In whole life	0.23	0.05	4.65	<0.0001 *	0.24	0.05	4.95	<0.0001 *
	In last year	0.30	0.07	4.15	<0.0001 *	0.08	0.02	3.71	0.0002 *
DUDIT questions: (chosen)									
Question 1		0.13	0.14	0.92	0.358				
Question 2		0.67	0.22	3.07	0.002 *				NS
Question 3		0.17	0.17	1.00	0.319				
Question 4		0.41	0.17	2.48	0.013 *				NS
Question 5		0.76	0.16	4.65	<0.0001 *				NS
Question 6		0.72	0.16	4.47	<0.0001 *				NS
DUDIT score		0.12	0.02	4.92	<0.0001 *				NS
DUDIT clinical groups		1.06	0.32	3.27	0.001 *				NS
Psychiatric treatment		0.73	0.34	2.14	0.033 *				NS

* *p* < 0.05.

**Table 11:** Univariate and multivariate analysis for parameters affecting the PQ-B subscale ‘distress score’. (β—β coefficient; SE—standard errors for coefficients; t—t value; *p*—*p* value; NS—non significant).

		Univariate Analysis				Multivariate Analysis			
		β	SE	t	*p*	β	SE	t	*p*
Sociodemographic									
Gender		−5.17	1.31	−3.95	0.0001 *	−5.7	1.28	−4.5	<0.00001 *
Age		−0.54	0.09	−5.77	<0.00001 *	−0.4	0.10	−3.8	0.0002 *
Heterosexual/Non-heterosexual		−4.23	1.53	−2.76	0.006 *				NS
In relationship		−4.77	1.34	−3.57	0.0004 *	−3.6	1.32	−2.8	0.006 *
Size of city of residence		−0.90	0.64	−1.42	0.1572				NS
Level of education		−5.30	1.10	−4.81	0.0000 *	−3.3	1.15	−2.9	0.004 *
Earnings		−1.84	0.34	−5.34	<0.0001 *				NS
Psychoactive substance									
Frequency of alcohol consumption		0.43	0.41	1.05	0.2964				
Age of first drug usage		−0.06	0.06	−1.08	0.2823				
Amount of types of drugs taken	In whole life	0.64	0.20	3.24	0.001 *	0.7	0.19	3.6	0.0004 *
	In last year	0.76	0.29	2.66	0.008 *				NS
DUDIT questions: (chosen)									
Question 1		0.22	0.56	0.39	0.6972				
Question 2		2.19	0.85	2.57	0.01 *				NS
Question 3		2.19	0.85	2.57	0.01 *	−2.4	0.74	−3.2	0.0015 *
Question 4		2.01	0.65	3.11	0.002 *				NS
Question 5		3.20	0.64	5.02	<0.00001 *				NS
Question 6		3.29	0.63	5.24	<0.00001 *				NS
DUDIT score		0.49	0.09	5.33	<0.00001 *	0.5	0.10	5.0	<0.00001 *
DUDIT clinical groups		3.59	1.27	2.82	0.005*				NS
Psychiatric treatment		4.73	1.32	3.58	0.0004	3.7	1.27	2.9	0.004 *

* *p* < 0.05.

## Data Availability

The data presented in this study are available on request from the corresponding author. The data are not publicly available due to privacy and legal reasons.
